# Characteristics associated with the transition to partial breastfeeding prior to 6 months of age: Data from seven sites in a birth cohort study

**DOI:** 10.1111/mcn.13166

**Published:** 2021-03-04

**Authors:** Stephanie A. Richard, Benjamin J. J. McCormick, Laura E. Murray‐Kolb, Crystal L. Patil, Ram K. Chandyo, Cloupas Mahopo, Bruna L. Maciel, Anuradha Bose, Mustafa Mahfuz, Ramya Ambikapathi, Maribel Paredes Olortegui, Laura E. Caulfield, the MAL‐ED Network Investigators

**Affiliations:** ^1^ Fogarty International Center National Institutes of Health Bethesda Maryland USA; ^2^ Infectious Disease Clinical Research Program Henry M Jackson Foundation for the Advancement of Military Medicine Bethesda Maryland USA; ^3^ Science Fish Limited Insch UK; ^4^ Department of Nutritional Sciences The Pennsylvania State University State College PA USA; ^5^ Department of Human Development and Nursing Science University of Illinois Chicago Illinois USA; ^6^ Kathmandu Medical College Kathmandu Nepal; ^7^ Department of Nutrition University of Venda Thohoyandou South Africa; ^8^ Department of Nutrition Federal University of Rio Grande do Norte Natal Brazil; ^9^ Division of Gastrointestinal Sciences Christian Medical College Vellore India; ^10^ Nutrition and Clinical Services Division icddr,b Dhaka Bangladesh; ^11^ Department of Public Health Purdue University West Lafayette Indiana USA; ^12^ AB Prisma Iquitos Peru; ^13^ The Department of International Health The Johns Hopkins Bloomberg School of Public Health Baltimore Maryland USA

**Keywords:** breastfeeding, food insecurity, infant feeding, infant growth, maternal depression

## Abstract

The WHO recommends exclusive breastfeeding for the first 6 months of life. However, the transition of the infants' diet to partial breastfeeding with the addition of animal milks and/or solids typically occurs earlier than this. Here, we explored factors associated with the timing of an early transition to partial breastfeeding across seven sites of a birth cohort study in which twice weekly information on infant feeding practices was collected. Infant (size, sex, illness and temperament), maternal (age, education, parity and depressive symptoms), breastfeeding initiation practices (time of initiation, colostrum and pre‐lacteal feeding) and household factors (food security, crowding, assets, income and resources) were considered. Three consecutive caregiver reports of feeding animal milks and/or solids (over a 10‐day period) were characterized as a transition to partial breastfeeding, and Cox proportional hazard models with time (in days) to partial breastfeeding were used to evaluate associations with both fixed and time‐varying characteristics. Overall, 1470 infants were included in this analysis. Median age of transition to partial breastfeeding ranged from 59 days (South Africa and Tanzania) to 178 days (Bangladesh). Overall, higher weight‐for‐length *z*‐scores were associated with later transitions to partial breastfeeding, as were food insecurity, and infant cough in the past 30 days. Maternal depressive symptoms (evaluated amongst 1227 infants from six sites) were associated with an earlier transition to partial breastfeeding. Relative thinness or heaviness within each site was related to breastfeeding transitions, as opposed to absolute *z*‐scores. Further research is needed to understand relationships between local perceptions of infant body size and decisions about breastfeeding.

**Key messages**

- Infants in resource‐constrained settings are often not fed according to recommendations, particularly 6 months of exclusive breastfeeding.
- A higher weight‐for‐length of the child given their age, recent coughing and food insecurity were associated with slower transition to partial breastfeeding.
- A higher maternal depressive symptom score was associated with earlier transition to partial breastfeeding before 6 months.

## 1 INTRODUCTION

The World Health Organization (WHO) recommends that infants are exclusively breastfed for 6 months (Kramer & Kakuma, 2012; Victora et al., 2016; World Health Organization [WHO], 2003); however, according to national surveys, only 37% of infants less than 6 months of age in low‐ and middle‐income countries (LMIC) are exclusively breastfed (Victora et al., 2016). Improved breastfeeding practices (i.e. exclusive breastfeeding for 6 months followed by continued breastfeeding until the child is 2 years of age) could potentially prevent over 800 000 annual child deaths in 75 high‐mortality LMICs (Victora et al., 2016) through reduced exposure to enteric pathogens and improved nutritional status.

Breastfeeding with the inclusion of other animal milks and solids is considered partial breastfeeding (Labbok & Krasovek, 1990). Mothers and other caregivers may transition to partial breastfeeding before 6 months for a variety of reasons, including factors related to the child (size, growth, morbidity and their behaviours [fussiness, appearing ready for foods]), the mother (age, education, parity, socioeconomic status, food security, depression, working outside of the home, perceived insufficient milk) and other contextual factors outside of the home including cultural norms (Kavle et al., 2017; Scott et al., 2009).

The Etiology, Risk Factors, and Interactions of Enteric Infections and Malnutrition and the Consequences for Child Health and Development (MAL‐ED) study is a longitudinal birth cohort that enrolled 2145 children in eight countries (MAL‐ED Network Investigators, 2014). The primary goal of the study was to describe the relationships amongst enteric infections, diet, gut function, and growth and development. Less than 60% of infants in six of the eight study sites were exclusively breastfed for the first month of life (Patil et al., 2015), and the median duration of exclusive breastfeeding across the sites was 33 days (Ambikapathi et al., 2016). In earlier analyses, infant feeding patterns during the first 2 years of life were found to influence growth in early childhood (MAL‐ED Network Investigators, 2017). The goal of this paper is to evaluate factors associated with the timing of transitions to partial breastfeeding before 6 months to inform exclusive breastfeeding promotion programmes in these and other low‐income settings.

## 2 METHODS

### 2.1 Data and setting

The MAL‐ED network included eight sites (Bangladesh [Dhaka: BGD], India [Vellore: INV], Nepal [Bhaktapur: NEB], Pakistan [Naushero Feroze: PKN], Brazil [Fortaleza: BRF], Peru [Loreto: PEL], South Africa [Venda: SAV] and Tanzania [Haydom: TZH]). Each site was to recruit and follow a cohort of 200 children to 24 months, and thus, enrolment varied by site based on projected loss to follow up (The MAL‐ED Network Investigators, 2014). Enrolment was staggered over a 2‐year period (during the overall period from 2009 to 2012) to account for seasonality in morbidity and pathogens. Infants were eligible for inclusion in the study if their birth weight or enrolment weight was ≥1500 g, they did not have a chronic illness at recruitment, they were a singleton birth, the family did not plan to move outside the community within 6 months, and their mother was at least 16 years of age. Caregivers provided written consent, and institutional ethical approval was obtained at each site. Further details are available elsewhere (The MAL‐ED Network Investigators, 2014). Here, we examine data from birth to 6 months of age.

At enrolment (within the first 17 days of life, median 7 days, inter‐quartile range 4 to 12 days), an interview was conducted to record child and family factors including the sex of the child, maternal age, parity, education and marital status. At that time, mothers reported specific details regarding the timing of breastfeeding initiation after delivery, whether colostrum was given, and pre‐lacteal feeding (Patil et al., 2015).

Thereafter, families were visited twice per week and asked about infant feeding practices in the preceding 24 h; specifically, caregivers were asked about the infant's consumption of breast milk, animal milk, formula, water, tea, fruit juice and other liquids or semi‐solids (Caulfield et al., 2014). Our primary outcome was the consistent reporting of partial breastfeeding, meaning that animal milks (including formula) and/or solids were added to the infant's diet alongside breast milk for three consecutive reports from the twice weekly breastfeeding data. We chose this definition based on prior work which described the episodic nature of exclusive breastfeeding across the study sites (Ambikapathi et al., 2016; Lee et al., 2014). During these same twice weekly visits, incidence of illness was recorded for all days since the prior visit. Illnesses included diarrhoea (≥3 loose stools/24 h), vomiting, coughing, acute lower‐respiratory infection (ALRI) and fever (Richard, Barrett, et al., 2014).

Infant weight and length were measured at enrolment and then monthly on the same birth day throughout the study (Richard, McCormick, et al., 2014). Age‐ and sex‐standardized *z*‐scores were derived from length and weight utilizing the WHO standards and methods (WHO, 2006). Personnel were trained on a common protocol prior to study enrolment and quality control measures were put in place (Richard, McCormick, et al., 2014); during the study, irreconcilable issues were found with the length data from PKN; therefore, the site was excluded from these analyses.

Food insecurity was assessed through the Household Food Insecurity Access Scale (HFIAS) at enrolment (Psaki et al., 2012; Swindale & Bilinsky, 2006), and households were considered to have food insecurity if they answered yes to any of the questions. At 6 months, households were surveyed about socioeconomic status including information on average household income, assets, crowding, and access to improved water and sanitation (as defined by the WHO) (Psaki et al., 2014).

Maternal depressive symptoms were captured using the Self‐Reporting Questionnaire at 1‐ and 6‐months postpartum (Beusenberg et al., 1994). The depressive symptoms data at each time point were subjected to psychometric analyses to ensure comparability across sites (Pendergast et al., 2014); because the 6‐month data as opposed to the 1‐month data were related to the outcome, and because the 6‐month data from BRF were not found comparable with the other sites, two analyses were run, with the maternal depressive symptom data (excluding BRF) or without (including BRF).

Child temperament was assessed at 6 months via caregiver report using the Infant Temperament Scale (ITS). Psychometric analyses supported the validity of an approach temperament factor across the eight sites (Pendergast et al., 2018). Approach temperament assesses an infant's tendency to move toward or engage in pleasurable or rewarding stimuli. Our assessment of this dimension of temperament reflected approach towards both social and physical stimuli. We used this as our best measure of infant behaviours, which might affect infant feeding decision‐making.

### 2.2 Statistical analysis

As noted above, we considered the report of feeding animal milks and/or solids on three consecutive visits prior to 6 months to indicate a transition to partial breastfeeding. The first instance of this transition was considered the ‘event’ for the purposes of a Cox proportional hazard model.

Cox proportional hazards models were constructed for individual variables and multivariable models. All models included site as a strata to account for differences in baseline hazards between sites. The home visits in which infant feeding and morbidity data were recorded were matched to the closest prior anthropometric measurement. This data structure allowed for time‐varying incidence of illness and the child's weight‐for‐length *z*‐score (WLZ) with monthly intervals (the anthropometry collection schedule) for months in which partial feeding was or was not initiated. Those not transitioning before 180 days (276/1470, or 18.8%) were censored. To put variables on a comparable scale, continuous variables, except for WLZ, were scaled to mean zero and standard deviation one across the MAL‐ED sites: average household income (log_10_ transformed first), the number of people per room, maternal age, maternal years of education, and depressive symptom scores at 6 months postpartum. Odds ratios for these variables therefore reflect the consequence of a one standard deviation change in the variable. The multivariable model was further subjected to backward stepwise selection [minimizing the Akaike Information Criterion (AIC)] to minimize over‐fitting. The models were globally proportional; however, WLZ was not (*χ* ^2^ 5.06, *p* = 0.02), largely due to the increase in WLZ observed (in all sites) over the first 2 months. Because field operations could result in shorter or longer than monthly periods between anthropometric measures, sensitivity analyses were conducted to examine the interval (median of 13 days, IQR 6 to 21) between the preceding anthropometry and the change in feeding; the model coefficients presented were found robust to weighting the regression by the interval even considering different weighting schemes.

## 3 RESULTS

Characteristics of the infants and households by site are shown in Table 1. Information on breastfeeding practices and relevant covariates through 6 months of age were available for 1470/1868 (78%) infants (Figure S1). Mothers were, on average, between 24 and 28 years old across the sites and had a median of between five (BGD) and 10 (SAV) years of education. Mothers depressive symptoms were infrequently reported at post‐partum or at 6 months (scores were typically <4 out of a possible 16). All study sites were considered low‐resource communities, but mean household incomes varied considerably across sites as did access to improved (i.e. reliably clean) water and sanitation. Children at the sites were, on average, between −0.6 and −1 length‐for‐age *z*‐scores (LAZ) at enrolment, whereas the enrolment weight‐for‐length *z*‐scores (WLZ) were more varied (−1.1 to 0.8) across the sites. The children in this study were most often the first or second child of the mother, except for TZH, which had a median birth order of four.

**TABLE 1:** Selected characteristics of the MAL‐ED cohort

	BGD	BRF	INV	NEB	PEL	SAV	TZH
	(*n* = 221)	(*n* = 176)	(*n* = 221)	(*n* = 207)	(*n* = 260)	(*n* = 157)	(*n* = 228)
*Median (IQR)*							
Age at transition from full breastfeeding^a^ (days)	176 (120, 192)	122 (69, 167)	124 (91, 148)	118 (51, 157)	172 (76, 191)	64 (39, 90)	58 (34, 85)
Length‐for‐age at enrolment (*z*‐score)	−0.97 (−1.72, −0.41)	−0.72 (−1.31, −0.2)	−1.02 (−1.65, −0.28)	−0.62 (−1.29, 0.12)	−0.88 (−1.52, −0.35)	−0.65 (−1.33, −0.1)	−0.97 (−1.68, −0.3)
Weight‐for‐age at enrolment (*z*‐score)	−1.3 (−1.88, −0.63)	−0.15 (−0.77, 0.42)	−1.21 (−1.81, −0.52)	−0.74 (−1.31, −0.26)	−0.59 (−1.11, −0.05)	−0.29 (−0.89, 0.25)	−0.15 (−0.72, 0.51)
Weight‐for‐length at enrolment (*z*‐score)	−0.91 (−1.64, −0.29)	0.5 (−0.44, 1.28)	−1.1 (−1.79, −0.44)	−0.89 (−1.49, −0.3)	−0.06 (−0.75, 0.56)	0.04 (−0.77, 0.83)	0.84 (0.03, 1.43)
Number (%) < 2500 g at enrolment	76 (34%)	7 (4%)	42 (19%)	20 (10%)	27 (10%)	15 (10%)	9 (4%)
Infant temperament score (0–52)	20 (17, 26)	17 (14, 22)	12 (11, 18)	23 (19, 26)	28 (24, 31)	23 (18, 27)	31 (28, 33)
Maternal age (years)	25 (21, 28)	25 (21, 28)	24 (21, 27)	26 (24, 29)	23.5 (19, 28)	26 (21, 33)	28 (23, 33)
Number of births (count)	2 (1, 3)	2 (1, 3)	2 (1, 3)	2 (1, 2)	2 (1, 4)	2 (1, 4)	4 (2, 6)
Maternal education (years)	5 (2, 7)	9 (7, 12)	8 (5, 9)	9 (5, 10)	8 (6, 10)	10 (9, 12)	7 (3, 7)
Maternal depressive symptoms (6 months, 16‐point scale)	5 (2, 9)		4 (1, 7)	4 (2, 7)	2 (1, 4)	2 (1, 4)	1 (0, 4)
Household monthly income (US$)	105 (72, 142)	345 (306, 395)	64.2 (45, 103)	138 (101, 208)	133 (104, 175)	191 (120, 292)	14 (7, 30)
People/room	3 (3, 4)	1.23 (0.96, 1.6)	3 (2, 4)	1.5 (1.14, 2.5)	1.67 (1.25, 2.33)	1.25 (0.89, 1.6)	2 (1.29, 2.67)
*N(%)*							
Sex (male)	106 (48)	82 (47)	98 (44)	111 (54)	141 (54)	78 (50)	113 (50)
Breastfeeding initiated <1 h	138 (62)	80 (45)	134 (61)	84 (41)	194 (75)	93 (59)	188 (82)
Fed colostrum	216 (98)	173 (98)	202 (91)	200 (97)	254 (98)	153 (97)	208 (91)
Pre‐lacteal feeding	28 (13)	11 (6)	27 (12)	37 (18)	22 (8)	3 (2)	10 (4)
Access to improved sanitation	221 (100)	173 (98)	104 (47)	206 (100)	66 (25)	153 (97)	32 (14)
Access to improved water	221 (100)	176 (100)	221 (100)	207 (100)	240 (92)	138 (88)	74 (32)
Any food insecurity	58 (26)	156 (89)	66 (30)	27 (13)	223 (86)	73 (46)	72 (32)

^a^ Defined as the first consistent (three consecutive visits) report of feeding animal milks or solids to a breastfed infant. Sites: BGD: Bangladesh—Dhaka; BRF: Brazil—Fortaleza; INV: India—Vellore; NEB: Nepal—Bhaktapur; PEL – Loreto; SAV: South Africa—Venda; TZH: Tanzania—Haydom.

**FIGURE 1:**
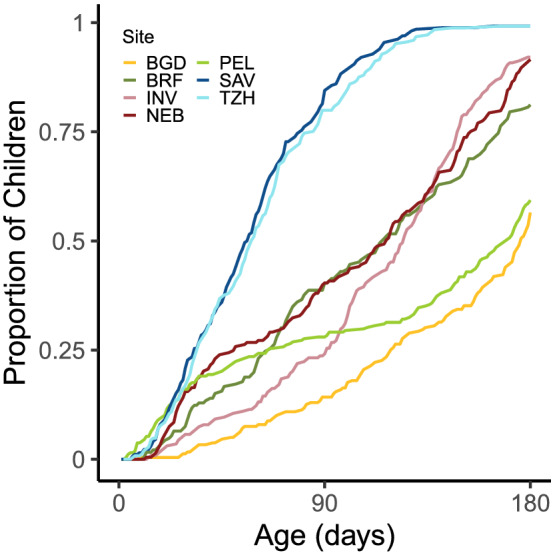
Cumulative proportion of infants by age at which they were transitioned to partial breastfeeding (three consecutive visits at which animal milks and/or solids were reported). Sites: BGD, Dhaka, Bangladesh; INV, Vellore, India; NEB, Bhaktapur, Nepal; BRF, Fortaleza, Brazil; PEL, Loreto, Peru; SAV, Venda, South Africa; TZH, Haydom, Tanzania

Because we were able to collect information on infant feeding twice weekly, we were able to characterize when this transition from exclusive to partial breastfeeding occurred. The median age (i.e. 50% of children) at which other milks or solid foods were consistently fed differs by site, with median ages of less than 2 months for SAV and TZH and ages near 6 months for BGD and PEL. The timing of the transition to partial breastfeeding is illustrated in Figure 1. Overall, of 1470 infants, 1190 (81%) transitioned from full breastfeeding before 180 days by our definition of three consecutive reports of partial or no breastfeeding. Of these, 38.7% had one prior report and another 25.6% had two prior reports of partial or no breastfeeding before the transition. All 280 of those who did not transition according to our definition (three consecutive visits) reported milk or solids (with a median of four introductions), but those introductions were not reported for three consecutive visits during the first 6 months of life. Only 9.6% of infants across the sites were weaned during the time period.

**FIGURE 2:**
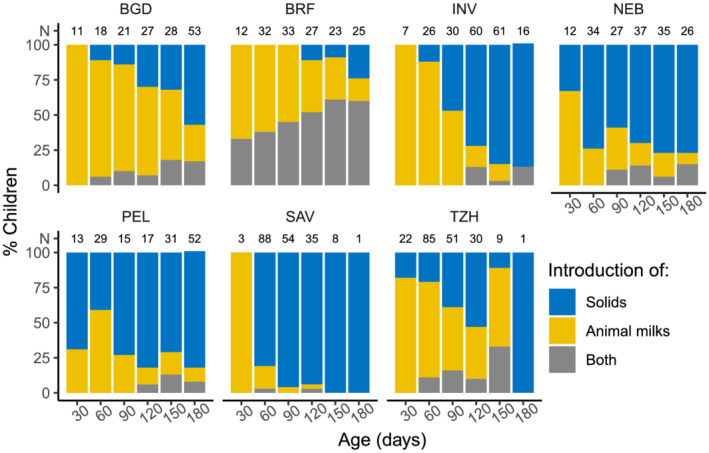
Percent of infants who introduced milks, solids, or both milks and solids in each month, considering those children transitioned to partial breastfeeding during each 30‐day period (N at top of columns). Sites: BGD, Dhaka, Bangladesh; INV, Vellore, India; NEB, Bhaktapur, Nepal; BRF, Fortaleza, Brazil; PEL, Loreto, Peru; SAV, Venda, South Africa; TZH, Haydom, Tanzania

Presented in Table 2 are the non‐breastmilk foods, specifically solids, formula, milks and a combination of these liquids and solids, introduced when partial breastfeeding began (reported over three consecutive visits). Amongst these children, solid foods were most often introduced, except for TZH where animal milks were the most frequently reported item introduced and BRF, where both animal milks and solid foods were frequently introduced together. As shown, commercial formula was reported in all sites except TZH. The solid foods introduced consisted primarily of cereals or grains, but root crops, legumes and banana were also fed (Table S1). The non‐breast milk foods introduced varied by infant age at occurrence (Figure 2). In the first month, if non‐breast milk foods were introduced, the introduced item was likely to be animal milks, except in PEL where they received solids. In month two, one‐third to one‐half of caregivers in SAV and TZH transitioned to partial breastfeeding, consisting of solids in SAV and animal milks in TZH.

**TABLE 2:** Category of non‐breast milk food fed to children when they were consistently fed other milks or solids on three consecutive visits prior to 6 months of age

Site^a^	Solids	Formula	Milks	Milks + solids	Formula + solids
BGD	103 (46%)	65 (29%)	24 (11%)	24 (11%)	9 (4%)
BRF	21 (12%)	53 (30%)	19 (11%)	85 (47%)	1 (1%)
INV	138 (62%)	0 (0%)	74 (33%)	12 (5%)	0 (0%)
NEB	145 (70%)	36 (17%)	9 (4%)	14 (7%)	3 (1%)
PEL	174 (70%)	22 (9%)	35 (14%)	16 (6%)	0 (0%)
SAV	132 (82%)	26 (16%)	0 (0%)	0 (0%)	3 (2%)
TZH	62 (26%)	0 (0%)	150 (64%)	24 (10%)	0 (0%)

^a^ BGD: Bangladesh—Dhaka; BRF: Brazil—Fortaleza; INV: India—Vellore; NEB: Nepal—Bhaktapur; PEL – Loreto; SAV: South Africa—Venda; TZH: Tanzania—Haydom.

Bivariate odds ratios for factors influencing the transition to partial breastfeeding (with site included as a fixed effect) are shown in Table 3. Of the morbidity variables considered, only reported cough over the prior 30 days was associated with the outcome. All variables tended to have small effect sizes, with the time‐invariant variables contributing to the baseline risk of transitioning to partial breastfeeding and the time‐varying factors, WLZ and presence of coughing, reflecting a changing risk over time. Of the child characteristics, WLZ and presence of coughing in the last 30 days were associated with decreased risk of partial breastfeeding. For WLZ, a one SD greater WLZ was associated with 16% lower likelihood of partial breastfeeding. Of the household factors, the reporting of food insecurity was associated with 18% lower likelihood of partial breastfeeding. As shown in bivariate analyses, factors associated with breastfeeding initiation and early practices were not associated with risk of transitioning to partial breastfeeding before 6 months.

**TABLE 3:** Hazard ratios for transition to partial breastfeeding prior to 6 months, based on Cox proportional hazards model in the MAL‐ED cohort study

		Bivariate	Stepwise multivariable models	
			Maternal depressive symptoms	
			Including (*n* = 1227)	Excluding (*n* = 1470)
Term	Unit	Hazard ratio (95% confidence interval)		
Sex	Male	1.11 (0.99, 1.24)	1.1 (0.97, 1.26)	***1.15 (1.02, 1.29)***
Length‐for‐age at enrolment	*z*‐score	0.95 (0.91, 1.01)	0.94 (0.89, 1)	0.94 (0.89, 1)
Weight‐for‐length^b^	*z*‐score	***0.84 (0.8, 0.88)***	***0.84 (0.79, 0.89)***	***0.83 (0.79, 0.87)***
Breastfeeding initiated <1 h	Yes	0.96 (0.85, 1.09)		
Fed colostrum	Yes	1.04 (0.78, 1.37)		
Pre‐lacteal feeding	Yes	1.14 (0.94, 1.4)		1.18 (0.96, 1.44)
Infant temperament	SD (count)	1.01 (0.93, 1.09)		
Cough in past 30 days^a^	Yes	***0.86 (0.76, 0.96)***	***0.85 (0.76, 0.96)***	***0.87 (0.77, 0.98)***
Diarrhoea in past 30 days^b^	Yes	0.96 (0.84, 1.11)		
Vomit in past 30 days^b^	Yes	0.99 (0.84, 1.15)		
Maternal age^a^	SD (years)	1.02 (0.97, 1.08)		
Number of births^a^	SD(n)	1.02 (0.96, 1.08)	1.06 (0.99, 1.14)	1.05 (0.98, 1.12)
Maternal education^a^	SD (years)	1.03 (0.97, 1.1)		
Maternal depressive symptoms at 1 month^a^	SD (count)	1 (0.98, 1.02)		
Maternal depressive symptoms at 6 months^a^	SD (count)	1.06 (0.99, 1.13)	***1.1 (1.02, 1.18)***	
Monthly household income^a^	SD (log10 US$)	1.04 (0.96, 1.12)		
People/room^a^	SD (count)	0.95 (0.89, 1.02)	0.92 (0.84, 1)	0.94 (0.87, 1.02)
Access to improved sanitation	Yes	1.08 (0.9, 1.3)		
Any food insecurity	Yes	***0.82 (0.72, 0.94)***	***0.76 (0.65, 0.88)***	***0.81 (0.71, 0.93)***

*Note*: Two models are shown, with or without maternal depressive symptoms. The model with depressive symptoms excluded data from the Brazilian site. All of the models include site as a strata to account for differences in baseline hazards between sites. Coefficients that do not include one in the 95% confidence interval.

^a^ Variables scaled to mean 0, SD 1.

^b^ Time‐varying variables, that is, measured at each day, whereas all other variables were measured once and change the baseline odds of ‘failure’. Site was included as a fixed effect in all models.

We present two multivariable regression models that adjusted for site in order to consider the influence of maternal depressive symptoms measured at 6 months along with other factors associated with risk of partial breastfeeding (Table 3). As mentioned earlier, the 6‐month as opposed to the 1‐month assessment of depressive symptoms was found to be associated with the transition to partial breastfeeding and the use of the 6‐month assessment necessitated exclusion of the BRF data. A 1 SD (3.5) above the mean maternal depressive symptoms score at 6 months was associated with a 10% greater likelihood of partial breastfeeding. Including maternal depressive symptoms, and therefore excluding BRF site data, did not yield significant differences to the risk estimates for coughing, WLZ or household food insecurity. However, in the model including the BRF site data, males were 15% more likely to be transitioned to partial breastfeeding. The hazard ratios for individual sites from each of these models are shown in Figures S2 and S3.

**FIGURE 3:**
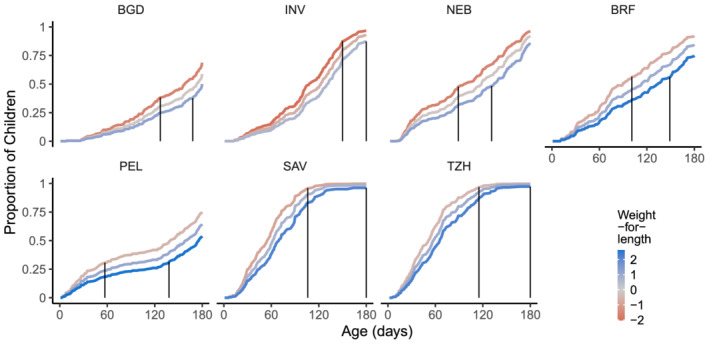
Proportion of infants, by age, at each of six MAL‐ED sites who transitioned to partial breastfeeding, stratified by weight‐for‐length *z*‐scores (WLZ). WLZ was incorporated into the model as a time‐varying variable; for illustrative purposes, a constant weight‐for‐length *z*‐score was assumed taking the site‐specific 10th, 50th or 90th percentile (pooled across all ages at each site). The vertical lines indicate the maximum difference in days between the 10th and 90th WLZ percentiles to reach the same proportion of infants with partial breastfeeding

The role of WLZ is noteworthy because the associations with feeding behaviour decisions change over time. Overall, mean WLZ increased from −0.34 at enrolment to 0.40 at 2 months, and in each site, mean WLZ increased from enrolment to 2 months and then plateaued or declined slightly through 6 months. To illustrate the changing likelihood of transitioning to partial breastfeeding associated with infant WLZ, we used the parameters from the model excluding maternal depressive symptoms (thus, including BRF) and depicted risks associated with (site‐specific) 10th, 50th, and 90th percentiles (Figure 3). After the first several weeks, a pattern emerges whereby lower WLZ is associated with having animal milks and/or solids introduced. The timing and nature vary by site, but the consistency of the pattern is evident and reflects relative heaviness/thinness within a site—rather than specific WHZ scores (e.g. −1 or −2 SD). Vertical lines are drawn in each figure to indicate the maximum age difference in the predicted transition to partial breastfeeding curves assuming an infant at the 10th or 90th percentile WLZ at each site; for example, the difference is greatest for PEL between 2 and 4 months, with an estimated 80‐day earlier transition to partial breastfeeding associated with WLZ of 0 (PEL 10th percentile) as compared with a WLZ of 2.5 (PEL 90th percentile). In NEB, between 3 and 4 months, a difference of 40 days is shown comparing an infant with WLZ of −1.5 (NEB 10th percentile) with the one with WLZ of 1 (NEB 90th percentile). For these illustrations, all other variables were held constant at their mean value for the respective site.

Using this same approach and the model including maternal depressive symptoms, figures were developed for the other factors in the model (Figures S4A–C). Mothers at their site‐specific 90th percentile for depressive symptoms at 6 months transitioned to partial breastfeeding earlier than those at the 10th percentile, with differences ranging from 5 days in SAV to 20 days in BGD, PEL and NEB (Figure S4C).

## 4 DISCUSSION

Many of the infants in the MAL‐ED cohort were not exclusively breastfed for the recommended 6 months. Because breastfeeding practices may temporarily vary depending on factors, including infant illness, or maternal work demands and travel, we chose to identify the transition to partial breastfeeding when the feeding of other milks or solids was reported on three consecutive occasions during twice weekly home visits. Across the sites, the transition to partial breastfeeding was associated negatively with child size, cough and food insecurity and positively with maternal depressive symptoms.

Infants with a lower WLZ were more likely to experience an earlier transition to partial breastfeeding during the first 6 months, even after adjusting for socioeconomic status and other factors. This suggests that mothers may alter their feeding practices according to how they perceive their infant to be growing, and, if they are perceived to be thinner than other infants in their setting, may introduce additional items into their diet. Although the difference in likelihood related to a 1 SD difference in WLZ is small, the associations are consistent across the seven diverse infant populations studied. Kramer et al. (2011) in a study in Belarus found that lower WLZ was associated with the introduction of non‐breast milk foods before 6 months, and Adair et al. (1993) evaluated categories of infant ponderal index (weight (g)/length (cm)^3^) and found ponderal index to be negatively associated with the transition to partial breastfeeding at 4 months in the Philippines. Our results extend those findings by demonstrating that the associations exist across seven different cultural settings and associated infant feeding norms and with varied yet overlapping distributions of infant WLZ. In studies of the transition away from exclusive or full breastfeeding, mothers may report perceived insufficient milk and relate this to smaller as well as larger infant size, or more general concerns about infant growth, health and behaviours (crying, fussiness and sleeping patterns) (Balogun et al., 2015; Kavle et al., 2017); however, these factors are considered amongst other socioeconomic and maternal factors associated with decision‐making.

In our analyses, the only maternal factor associated with the transition to partial breastfeeding was maternal depressive symptoms, assessed at 6 months postpartum. The likelihood was 10% higher over time for those with 1 SD more depressive symptoms. There is much interest in postpartum depression and how it may negatively impact breastfeeding, other care practices and child growth, and although research suggests that depression is associated with shorter duration of exclusive breastfeeding, studies from LMIC are limited (Dadi et al., 2020; Dennis & McQueen, 2009; Rahman et al., 2016; Surkan et al., 2011). We did not find associations with our assessment at 1‐month postpartum but did find associations at 6 months. In our psychometric analyses of the SRQ, we found that somatic symptoms (such as difficulty sleeping, headaches and digestive difficulties) were not reflective of internalizing symptoms (Pendergast et al., 2014). This may be especially true soon after childbirth (month one), and therefore, the scores at 6 months may more accurately reflect depression during the postpartum period in these settings. Research suggests higher prevalence of postpartum depression across LMIC, and our findings are noteworthy because the average number of depressive symptoms reported was low, indicating that even mild depressive symptoms may affect decisions around infant feeding. Researchers have raised concerns about bias due to potential reverse causality with respect to maternal depression and outcomes such as poor child growth; as our assessment of maternal depressive symptoms was at 6 months, we cannot be sure of the directionality of the association between maternal depressive symptoms and the timing of the transition to partial breastfeeding during a period in which exclusive breastfeeding is recommended. Such concerns have also been raised with respect to evaluations of breastfeeding practices and growth (Kramer et al., 2011, 2018), and our results support this need for caution as we assessed WLZ on a monthly basis and report concurrent and prospective risk of transitioning to partial breastfeeding prior to 6 months.

We conducted these analyses to better understand factors associated with the early transition to partial breastfeeding in our study populations. That being said, the primary limitation of these analyses is that the study was not designed to understand decision‐making regarding infant feeding practices. Many variables of interest were not collected; for example, child behavioural cues (beyond approach temperament), maternal intentions, maternal work or schooling, and reported reasons for the change in feeding pattern. For example, a semi‐structured interview would have allowed us to further explore reasons for the introduction of other milks and solids prior to 6 months as well as the overall observed pattern. That said, the SAV site performed a qualitative study to explore factors influencing infant feeding decisions and identified that mothers going back to work or school was a strong factor influencing the introduction of non‐breast milk foods, in addition to perceived breast milk insufficiency and community influences (Mushaphi et al., 2017). A focused analysis of the data from TZH revealed that the decision to introduce animal milk or solids was associated with ownership of cattle (Hanselman et al., 2018). Strengths of our study include our twice weekly surveillance of feeding practices which allowed us to identify consistent shifts in feeding pattern, and the detailed psychometric analyses of data collected to characterize maternal depressive symptoms and aspects of child development. Psychometric analyses were performed to substantiate the validity of data pooling, but a corollary of this rigorous approach is the need to exclude sites from analyses, leading us to present results with or without the BRF data in the model. We also have limited information regarding the paediatric primary care settings at each site and whether, for example, infant growth was assessed or discussions occurred which led to a change in feeding patterns. Families in the sites in PEL, BRF, INV and BGD had access to a relatively well‐developed care setting, whereas those in NEB, SAV and TZH had little to no programmatic activity. Because our results regarding variation in WLZ and partial breastfeeding are distribution specific and do not support a generalized cut‐point, such as −2, it is more likely that cultural norms or familial and maternal perceptions of size led to the associations observed. Finally, we could not assess gestational age in our study, and therefore, we cannot identify infants born preterm; thus, the WLZ of preterm infants in our sample will be misclassified to an unknown degree, and the influence of gestational age on feeding decisions in these sites is not known.

## 5 CONCLUSIONS

Children in resource‐constrained settings are often not fed according to recommendations, particularly 6 months of exclusive breastfeeding. In the populations studied here, exclusive breastfeeding typically lasted approximately a month, and then animal milks were introduced and quickly followed by solids. The timing of this change in feeding pattern ranged from around 2 to 6 months depending on the population, but it was consistently slowed by a higher weight‐for‐length of the child given their age, recent coughing, and food insecurity, but sped up by more maternal depressive symptoms. More targeted research is needed to understand caregiver perceptions of child size within the cultural norms of the community and decision making about infant feeding.

## CONFLICTS OF INTEREST

The authors declare that they have no conflicts of interest.

## CONTRIBUTIONS

The authors responsibilities were as follows: SAR, BJJM and LEC designed the analysis; SAR and BJJM performed the statistical analysis; SAR, LEMK, BJJM and LEC contributed to the analysis and interpretation of results; RA, AB, MM, RC, MI, BLM and MPO were members of the Nutrition Technical Subcommittee and also conducted the research; SAR, BJJM, LEMK and LEC wrote the manuscript; SAR and LEC have primary responsibility for final content; and all authors read and approved the final manuscript.

6

## Supporting information


**Data S1.** Supporting Information
**Figure S1.** Flow chart of infants included in analyses. Numbers are given for the analytic sample, and below, the sample size excluding the maternal depressive symptoms data. BGD: Bangladesh—Dhaka; INV: India—Vellore; NEB: Nepal—Bhaktapur; BRF: Brazil—Fortaleza; PEL: Peru – Loreto; SAV: South Africa—Venda; TZH: Tanzania—Haydom.
**Figure S2:** Mean hazard ratios and 95% confidence intervals overall (black) and by site (colours) for the stepwise selection of the multivariable Cox model for transition to partial breastfeeding that included maternal depressive symptoms (and excluded Brazilian data). BGD: Bangladesh—Dhaka; INV: India—Vellore; NEB: Nepal—Bhaktapur; BRF: Brazil—Fortaleza; PEL: Peru – Loreto; SAV: South Africa—Venda; TZH: Tanzania—Haydom.
**Figure S3:** Mean hazard ratios and 95% confidence intervals overall (black) and by site (colours) for the stepwise selection of the multivariable Cox model for transition to partial breastfeeding that excluded maternal depressive symptoms (and included Brazilian data). BGD: Bangladesh—Dhaka; INV: India—Vellore; NEB: Nepal—Bhaktapur; BRF: Brazil—Fortaleza; PEL: Peru – Loreto; South Africa—Venda; TZH: Tanzania—Haydom.
**Figure S4 (A)** Proportion of infants, by age, at each of six MAL‐ED sites who transitioned to partial breastfeeding, stratified by cough in the preceding 30d. Cough was incorporated into the model as a time‐varying variable; for illustrative purposes, a constant Y/N was assumed. The vertical lines indicate the maximum difference in days to reach the same proportion of infants with partial breastfeeding. BGD: Bangladesh—Dhaka; INV: India—Vellore; NEB: Nepal—Bhaktapur; PEL: Peru – Loreto; SAV: South Africa—Venda; TZH: Tanzania—Haydom.
**Figure S4 (B)** Proportion of infants, by age, at each of six MAL‐ED sites who transitioned to partial breastfeeding, stratified by food insecurity (measured at baseline). The vertical lines indicate the maximum difference in days to reach the same proportion of infants with partial breastfeeding. BGD: Bangladesh—Dhaka; INV: India—Vellore; NEB: Nepal—Bhaktapur; PEL: Peru – Loreto; SAV: South Africa—Venda; TZH: Tanzania—Haydom.
**Figure S4 (C)** Proportion of infants, by age, at each of six MAL‐ED sites who transitioned to partial breastfeeding, stratified by maternal depressive symptoms (measured at six months). A constant depressive score was assumed taking the site‐specific 10^th^, 50^th^ or 90^th^ percentile (pooled across all ages for a given site). The vertical lines indicate the maximum difference in days to reach the same proportion of infants with partial breastfeeding. BGD: Bangladesh—Dhaka; INV: India—Vellore; NEB: Nepal—Bhaktapur; PEL: Peru – Loreto; SAV: South Africa—Venda; TZH: Tanzania—Haydom.
**Table S1.** Type of solid food given to the infants when they were consistently fed milks and/or solid foods on three consecutive visits. Multiple types of solid foods may have been reported, therefore the percentages, if summed, exceed 100%.

## Data Availability

Data can be requested at the following website: https://clinepidb.org/ce/app/record/dataset/DS_5c41b87221.
