# Supplementary material for: Characteristics associated with the transition to partial breastfeeding prior to 6 months of age: Data from seven sites in a birth cohort study
Source: Matern Child Nutr. 2021 Mar 4;17(3):e13166. doi: 10.1111/mcn.13166 (PMC8189203; doi:10.1111/mcn.13166)
Supplement: Supplementary file 1 — Data S1. Supporting Information Figure S1. Flow chart of infants included in analyses. Numbers are given for the analytic sample, and below, the sample size excluding the maternal depressive symptoms data. BGD: Bangladesh—Dhaka; INV: India—Vellore; NEB: Nepal—Bhaktapur; BRF: Brazil—Fortaleza; PEL: Peru – Loreto; SAV: South Africa—Venda; TZH: Tanzania—Haydom. Figure S2: Mean hazard ratios and 95% confidence intervals overall (black) and by site (colours) for the stepwise selection of the multivariable Cox model for transition to partial breastfeeding that included maternal depressive symptoms (and excluded Brazilian data). BGD: Bangladesh—Dhaka; INV: India—Vellore; NEB: Nepal—Bhaktapur; BRF: Brazil—Fortaleza; PEL: Peru – Loreto; SAV: South Africa—Venda; TZH: Tanzania—Haydom. Figure S3: Mean hazard ratios and 95% confidence intervals overall (black) and by site (colours) for the stepwise selection of the multivariable Cox model for transition to partial breastfeeding that excluded maternal depressive symptoms (and included Brazilian data). BGD: Bangladesh—Dhaka; INV: India—Vellore; NEB: Nepal—Bhaktapur; BRF: Brazil—Fortaleza; PEL: Peru – Loreto; South Africa—Venda; TZH: Tanzania—Haydom. Figure S4 (A) Proportion of infants, by age, at each of six MAL‐ED sites who transitioned to partial breastfeeding, stratified by cough in the preceding 30d. Cough was incorporated into the model as a time‐varying variable; for illustrative purposes, a constant Y/N was assumed. The vertical lines indicate the maximum difference in days to reach the same proportion of infants with partial breastfeeding. BGD: Bangladesh—Dhaka; INV: India—Vellore; NEB: Nepal—Bhaktapur; PEL: Peru – Loreto; SAV: South Africa—Venda; TZH: Tanzania—Haydom. Figure S4 (B) Proportion of infants, by age, at each of six MAL‐ED sites who transitioned to partial breastfeeding, stratified by food insecurity (measured at baseline). The vertical lines indicate the maximum difference in days to reach the same proportion [file MCN-17-e13166-s001.docx]

**Figure S1.** Flow chart of infants included in analyses. Numbers are given for the analytic sample, and below, the sample size excluding the maternal depressive symptoms data. BGD: Bangladesh—Dhaka; INV: India—Vellore; NEB: Nepal—Bhaktapur; BRF: Brazil—Fortaleza; PEL: Peru – Loreto; SAV: South Africa—Venda; TZH: Tanzania—Haydom.

**Table S1.** Type of solid food given to the infants when they were consistently fed milks and/or solid foods on three consecutive visits. Multiple types of solid foods may have been reported, therefore the percentages, if summed, exceed 100%.

| **Site^a^** | **# with solids** | **Cereals** | **Legumes** | **Root vegetables** | **Banana** |  |  |
| --- | --- | --- | --- | --- | --- | --- | --- |
| BGD | 136 | 124 (91%) | 20 (15%) | 20 (15%) | 18 (13%) |  |  |
| BRF | 107 | 93 (87%) | 0 (0%) | 29 (27%) | 7 (7%) |  |  |
| INV | 150 | 145 (97%) | 17 (11%) | 2 (1%) | 0 (0%) |  |  |
| NEB | 162 | 155 (96%) | 79 (49%) | 2 (1%) | 1 (1%) |  |  |
| PEL | 190 | 140 (74%) | 4 (2%) | 58 (31%) | 29 (15%) |  |  |
| SAV | 135 | 129 (96%) | 0 (0%) | 0 (0%) | 0 (0%) |  |  |
| TZH | 86 | 86 (100%) | 1 (1%) | 0 (0%) | 1 (1%) |  |  |

^a^BGD: Bangladesh—Dhaka; INV: India—Vellore; NEB: Nepal—Bhaktapur; BRF: Brazil—Fortaleza; PEL: Peru – Loreto; SAV: South Africa—Venda; TZH: Tanzania—Haydom

**Figure S2:** Mean hazard ratios and 95% confidence intervals overall (black) and by site (colors) for the stepwise selection of the multivariable Cox model for transition to partial breastfeeding that included maternal depressive symptoms (and excluded Brazilian data). BGD: Bangladesh—Dhaka; INV: India—Vellore; NEB: Nepal—Bhaktapur; BRF: Brazil—Fortaleza; PEL: Peru – Loreto; SAV: South Africa—Venda; TZH: Tanzania—Haydom.

**Figure S3:** Mean hazard ratios and 95% confidence intervals overall (black) and by site (colors) for the stepwise selection of the multivariable Cox model for transition to partial breastfeeding that excluded maternal depressive symptoms (and included Brazilian data). BGD: Bangladesh—Dhaka; INV: India—Vellore; NEB: Nepal—Bhaktapur; BRF: Brazil—Fortaleza; PEL: Peru – Loreto; South Africa—Venda; TZH: Tanzania—Haydom.

**Figure S4 (A)** Proportion of infants, by age, at each of six MAL-ED sites who transitioned to partial breastfeeding, stratified by cough in the preceding 30d. Cough was incorporated into the model as a time-varying variable; for illustrative purposes, a constant Y/N was assumed. The vertical lines indicate the maximum difference in days to reach the same proportion of infants with partial breastfeeding. BGD: Bangladesh—Dhaka; INV: India—Vellore; NEB: Nepal—Bhaktapur; PEL: Peru – Loreto; SAV: South Africa—Venda; TZH: Tanzania—Haydom.

**(B)** Proportion of infants, by age, at each of six MAL-ED sites who transitioned to partial breastfeeding, stratified by food insecurity (measured at baseline). The vertical lines indicate the maximum difference in days to reach the same proportion of infants with partial breastfeeding. BGD: Bangladesh—Dhaka; INV: India—Vellore; NEB: Nepal—Bhaktapur; PEL: Peru – Loreto; SAV: South Africa—Venda; TZH: Tanzania—Haydom.

**(C)** Proportion of infants, by age, at each of six MAL-ED sites who transitioned to partial breastfeeding, stratified by maternal depressive symptoms (measured at six months). A constant depressive score was assumed taking the site-specific 10^th^, 50^th^ or 90^th^ percentile (pooled across all ages for a given site). The vertical lines indicate the maximum difference in days to reach the same proportion of infants with partial breastfeeding. BGD: Bangladesh—Dhaka; INV: India—Vellore; NEB: Nepal—Bhaktapur; PEL: Peru – Loreto; SAV: South Africa—Venda; TZH: Tanzania—Haydom.
